# Cuticular fatty acids of *Galleria mellonella* (Lepidoptera) inhibit fungal enzymatic activities of pathogenic *Conidiobolus coronatus*

**DOI:** 10.1371/journal.pone.0192715

**Published:** 2018-03-08

**Authors:** Anna Katarzyna Wrońska, Mieczysława Irena Boguś, Emilia Włóka, Michalina Kazek, Agata Kaczmarek, Katarzyna Zalewska

**Affiliations:** 1 Witold Stefański Institute of Parasitology, Polish Academy of Sciences, Twarda, Warsaw, Poland; 2 BIOMIBO, Warsaw, Poland; University of Illinois, UNITED STATES

## Abstract

The entomopathogenic fungus *Conidiobolus coronatus* produces enzymes that may hydrolyze the cuticle of *Galleria mellonella*. Of these enzymes, elastase activity was the highest: this figure being 24 times higher than NAGase activity 553 times higher than chitinase activity and 1844 times higher than lipase activity. The present work examines the differences in the hydrolysis of cuticles taken from larvae, pupae and adults (thorax and wings), by *C*. *coronatus* enzymes. The cuticles of the larvae and adult thorax were the most susceptible to digestion by proteases and lipases. Moreover, the maximum concentration of free *N*-glucosamine was in the hydrolysis of *G*. *mellonella* thorax. These differences in the digestion of the various types of cuticle may result from differences in their composition. GC-MS analysis of the cuticular fatty acids isolated from pupae of *G*. *mellonella* confirmed the presence of C 8:0, C 9:0, C 12:0, C 14:0, C 15:0, C 16:1, C 16:0, C 17:0, C 18:1, C 18:0, with C 16:0 and C 18:0 being present in the highest concentrations. Additional fatty acids were found in extracts from *G*. *mellonella* imagines: C 10:0, C 13:0, C 20:0 and C 20:1, with a considerable dominance of C 16:0 and C 18:1. In larvae, C 16:0 and C 18:1 predominated. Statistically significant differences in concentration (p≤0.05) were found between the larvae, pupae and imago for each fatty acid. The qualitative and quantitative differences in the fatty acid composition of *G*. *mellonella* cuticle occurring throughout normal development might be responsible for the varied efficiency of fungal enzymes in degrading larval, pupal and adult cuticles.

## Introduction

Insect populations are naturally regulated by entomopathogenic fungi. At present, in the literature there are data indicating the possibility of using entomopathogenic fungi to control insect pests [1,2]. Their mode of entry into the insect body is based around two mechanisms: penetration of the cuticle by the growing hyphae or specialized infectious structures like appressoria or penetrant tubes, and by the enzymatic degradation of major cuticle components, including proteins, chitin and lipids [3,4]. The species-specific nature of the exoskeleton seems to be a decisive factor governing the sensitivity or resistance of various insect species to fungal infection [5]. The composition of the cuticle strongly influences conidial adherence and germination, resulting in the susceptibility to a fungal pathogen varying between species [4, 6, 7, 8].

The outer layers of the insect epicuticle contain lipids consisting mainly of aliphatic polar and non-polar compounds [9]. These substances are often involved in various types of chemical communication between species and between the different developmental stages (larvae, pupae, imago) of a single species [10, 11]. The lipid composition of the cuticle can also be used to assign insects to specific taxonomic groups [12, 13]. The components of epicuticular lipids are short-chain alcohols, wax esters (esters of long-chain alcohols and long-chain acids), esters of short-chain acids and, as well as ketones, aldehydes, oxoaldehydes, secondary alcohols, free fatty acids and acylglycerols [9, 12, 14]. The germination and differentiation of fungal spores can be modulated by cuticular fatty acids: they can have toxic or fungistatic effects on some pathogenic species or stimulatory effects on others [3,15].

The determination of cuticular fatty acid profiles will understand the mechanisms of insect susceptibility to fungal infection, and the field application of entomopathogenic fungi as biological control agents requires a better understanding of the physiological aspects of fungal growth, the nature of virulence factors and complex interactions between the fungal pathogen and the host cuticle. Our study examines the relationships between the free fatty acid composition of the cuticle and its susceptibility to digestion by fungal proteo-, chitino- and lipolytic enzymes. As a model system, the wax moth *G*. *mellonella* and entomopathogenic soil fungus *C*. *coronatus* were chosen. *C*. *coronatus* produces proteases, chitinases and lipases. This fungus induced 90% mortality in a *G*. *mellonella* host population [16, 17].

## Materials and methods

### Fungus

*Conidiobulus coronatus* (isolate number 3491), originally isolated from *Dendrolaelaps spp*., was obtained from the collection of Prof. Bałazy (Polish Academy of Sciences, Research Center for Agricultural and Forest Environment, Poznań). It was routinely maintained in 90 mm Petri dishes at 20°C with cyclic changes of light (L:D 12:12) on Sabouraud agar medium (SAM) with the addition of homogenized *Galleria mellonella* (Lepidoptera: Pyralidae) larvae to a final concentration of 10% wet weight. The sporulation and virulence of the SAM *C*. *coronatus* cultures was enhanced with the addition of homogenized *G*. *mellonella* larvae. Seven-day-old cultures were rinsed with sterile water to harvest the conidia, and 100 μl portions of the suspension, each containing ca 50 conidia (which were counted using the Thoma chamber), were used for inoculations.

In order to obtain the mixture of fungal enzymes hydrolyzing the insect cuticle, *C*. *coronatus* was cultivated at 20°C in 500 ml Erlenmeyer flasks containing 250 ml of minimal medium, as described by Bania et al. [16], but without shaking. Even very gentle shaking causes the mycelium to clump. Without shaking in the liquid culture mycelium is loose and filamentous. Three weeks after inoculation, the mycelia were removed by filtration through Whatman no. 1 filter paper. Cell-free filtrates were used for the *in vitro* hydrolysis of *G*. *mellonella* cuticle preparations.

### Insects

A culture of the wax moth, *G*. *mellonella* (Lepidoptera) was maintained and reared in temperature and humidity-controlled chambers (30°C, 70% r.h.) in constant darkness on an artificial diet [18]. Fully-grown larvae were collected before pupation, surface-sterilized and homogenized, and then used as a supplement in the fungal cultures. The larvae were also used in the virulence tests routinely performed after each fungus transfer [17]. Five-day-old last instar larvae (5DLL), two-day-old pupae (2DP) and two-day-old adults (2DA) served as cuticle donors and were used for lipid extractions.

### Infection of insects with *C*. *coronatus*

*G*. *mellonella* larvae (five-day-old last instar), pupae (two-day-old pupae) and adults (two-day-old adults) were exposed for 24 hours at a temperature of 30°C to fully-grown and sporulating *C*. *coronatus* colonies. Around 15 individuals were maintained in each Petri dish. A control group was formed of larvae exposed for 24 hours to sterile Sabouraud agar medium. There were 100 insects in both groups of insects, control and exposed to fungus. After exposure, the insects were transferred to new, clean Petri dishes with appropriate food (an artificial diet [18] and kept for one day. Following this 24-hour exposure to the fungus, one group of insects was collected immediately for examination (24-hour group) while the rest were left for another 24 hours before collection (48-hour group).

### Cuticle preparation

The *G*. *mellonella* larvae were anaesthetized by submersion in tap water for five to 10 minutes while the adults were immobilized on ice. The cuticle was dissected from the larvae and adult thoraces in ice-cold 10 mM Tris-HCl buffer at pH 7.0. The remnants of muscles, fat body and epidermis were precisely cleared away. The cocoons were regularly isolated from the colony, the pupae removed from them and left in glass jars until eclosion. The pupal cuticle was obtained by collecting empty exoskeletons. The wings were resected from frozen adults. All prepared cuticle pieces were washed three times in ice-cold 10 mM Tris-HCl buffer at pH 7.0, allowed to dry on an ice-cold towel and stored in -20°C until use.

### Enzymatic assays

The activities of elastase, *N*-acetylglucosaminidase (NAGase), chitinase and lipase were measured in the *C*. *coronatus* cell-free filtrates. The enzymatic activities were measured spectrophotometrically and spectrofluorimetrically (BioTek Synergy HT) in 96-well polystyrene plates using suitable synthetic substrates (all from Sigma, Poland). The activity of each enzyme was determined in four independent replicates. Elastolytic activity was measured using the chromogenic substrate *N*-Succinyl-Ala-Ala-Pro-Leu-p-Nitroanilide in a 100 mM Tris-HCl buffer containing 20 mM CaCl_2_, pH 8.0.

The reactions were performed in plate wells, each containing 2 μl of cell-free filtrate (which was thoroughly mixed for a minute) containing enzymes released by the fungus, 0.5 mM final substrate concentration, made up to a final volume of 200 μl with reaction buffer (Tris-HCl pH 7.0 or 10.0- depending on the substrate used). The experiment was conducted at 30°C. Progress curves were plotted during the course of the reaction after the substrate was added by spectrophotometric examination at A_410_. Chitobiosidase activity was measured using a 0.003 mM final concentration of fluorogenic substrate 4-Methylumbelliferyl β-D-N-N’-diacetylchitobioside in a 50 mM Tris-HCl buffer (pH 7.0). Fluorescence was read at Ex = 340 nm and Em = 450 nm. NAGase activity was measured using a 0.3 mM concentration of the chromogenic substrate 4-Nitrophenyl-*N*-acetyl-β-D-glucosaminide in a 10 mM Tris-HCl buffer (pH 7.0). Absorbance was read at 405 nm. Lipase activity was measured using a 0.01 mM final concentration of the fluorogenic substrate 4-Methylumbelliferyl oleate in a 50 mM Tris-HCl buffer, pH 10.0. Fluorescence was read at Ex = 360 nm and Em = 450 nm.

### Determination of protein concentration

Protein concentration was determined in the cell-free filtrate of *C*. *coronatus* with the Bio-Rad Protein Assay, which based on the method of Bradford. It involves the addition of an acidic dye (Coomassie^®^ Brilliant Blue) to protein solution, and subsequent measurement at 595 nm with a microplate reader. Absorbances were measured using BioTek Synergy HT. Bovine serum albumin (BSA) was used as the standard.

### Hydrolysis of the insect cuticle incubated with the cell-free filtrate of *C*. *coronatus*

The cuticle samples collected from the insects were divided into 50 mg portions, ground in liquid nitrogen and then washed four times with a 10 mM Tris-HCl buffer (pH 7.0). After washing the cuticles were dried. Following this, 10 mg of ground cuticle was suspended in 1ml of the 10mM Tris-HCl buffer (pH 7.0), and 800 μl of this sample was mixed with 228 μlof the *C*. *coronatus* cell-free filtrate containing elastase, NAGase, chitobiosidase and lipase. The reaction mixture was incubated for eight hours at 30°C. Afterwards, the reaction mixture was divided into 20 μl portions and immediately frozen to stop further hydrolysis. Two negative controls were added, one consisting of 1 mg of cuticle in the reaction buffer without the cell-free *C*. *coronatus* filtrate (C1), and the other of a buffer containing the cell-free filtrate of *C*. *coronatus* but without the insect cuticle (C2).

The products of cuticle hydrolysis by the fungal proteases were free amino acids; The samples and the controls were mixed with 0.1% Picrylsulfonic acid (Sigma) and read at A_340_. The absorbance of the negative controls was subtracted from the samples. The amounts of *N*-glucosamine released from the cuticle hydrolyzed by fungal chitinases were measured using the D-glucosamine Assay Kit (Megazyme) according to the producer’s manual. The concentrations of free fatty acids released by lipases were determined with the use of the EnzymChrom^™^ Free Fatty Acid Assay Kit (BioAssay Systems). Three independent replicates of all procedures were performed.

### Extraction of free fatty acids (FFAs)

Surface lipid components were extracted, separated and analyzed by GC-MS. The cuticular FFAs were extracted by method described previously [19] with modification. The applied volumes of solvents were modified for shorten time needed for evaporation and reduce losses during this process. Larvae, pupae and adult insects were extracted first for five minutes in 20 mL of petroleum ether (Merck Millipore) (extract I), and then for another five minutes in 20 mL of dichloromethane (Sigma) (extract II). Use of petroleum ether minimizes the possible extraction of internal lipids.

The extracts were placed in glass flasks and then evaporated under a stream of nitrogen. Table 1 includes the numbers of used insects at various developmental stages, as well as the dry masses of the extracts.

**Table 1:** Quantitative summary of the experiment: Numbers of used *Galleria mellonella* larvae, pupae and adults; masses of extracts.

Developmental stage	Total number of insects	Total masses of insects (g)	Total masses of ether extracts (mg/g of insects)	Total masses of dichloromethane extracts (mg/g of insects)
**Larvae (5DLL)**	12	2.16	0.52	0.63
**Pupae (2DP)**	14	1.58	0.77	0.79
**Adults (2DA)**	40	2.30	0.47	0.57

Extractions of cuticular lipids from larvae, pupae and adults were performed in triplicates (larvae N = 4 in each extraction, pupae N = 4–5, adults N = 13–14, respectively).

### Derivatization method

Derivatization was performed as described earlier [19]. Trimethylsilyl esters (TMS) of FFAs were obtained by the addition of 100 μl of BSTFA:TMCS mixture (99:1) (Sigma) to 1 mg of each sample, and then heating for one hour at 100°C. The TMSs of the fatty acids were then analyzed by GC/MS.

### GC/MS analyses

The analyses were carried out on a GCMS-QP2010 with mass detector (Shimadzu). Helium was used as the carrier gas, with a column head pressure of 65.2 kPa. The injection mode was split. A DB-5 MS (Zebron) column was used (thickness 0.25 μm, length 30 m, diameter 0.25 μm) The column oven temperature cycle was held at 80 °C for three minutes and then ramped from 80 °C to 310 °C at 4 °C/minute; the final temperature was then held for 10 minutes. The ion source temperature was 200 °C and the interface temperature was 310 °C. Split mode was used. Split ratio was 10.

All compounds were identified on the basis of fragmentation patterns and mass-to-charge ions of the TMS derivatives and the NIST 11 library. The mass spectrum of trimethylsilyl esters of fatty acids revealed the presence of the following ions: M+ (molecular ion), [M-15]+, and fragment ions at m/z 117, 129, 132 and 145. GC analysis employed the 19-methylarachidic acid (1 mg/ml; Sigma) as internal standard (IS). The contents were calculated from the relative peak areas that were compared to the IS peak area and expressed as the percentage (%, w/w) of total extracts. Response factors of one were assumed for all constituents.

### Statistics

The obtained results were tested using the parametric t-test and the analysis of variance (ANOVA), with the results being significant at p≤0.05. STATISTICA software (StatSoftPolska) was used for the evaluation of data normality (skewness, kurtosis), statistical significance, and for the calculation of correlation coefficients. The p-values and F-values obtained in the one-way ANOVA tests are presented in the Results section. For *post hoc* analysis, Tukey’s test was used. Each test was performed separately.

## Results

### The sensitivity of insects to fungal infection

**Fig 1:**
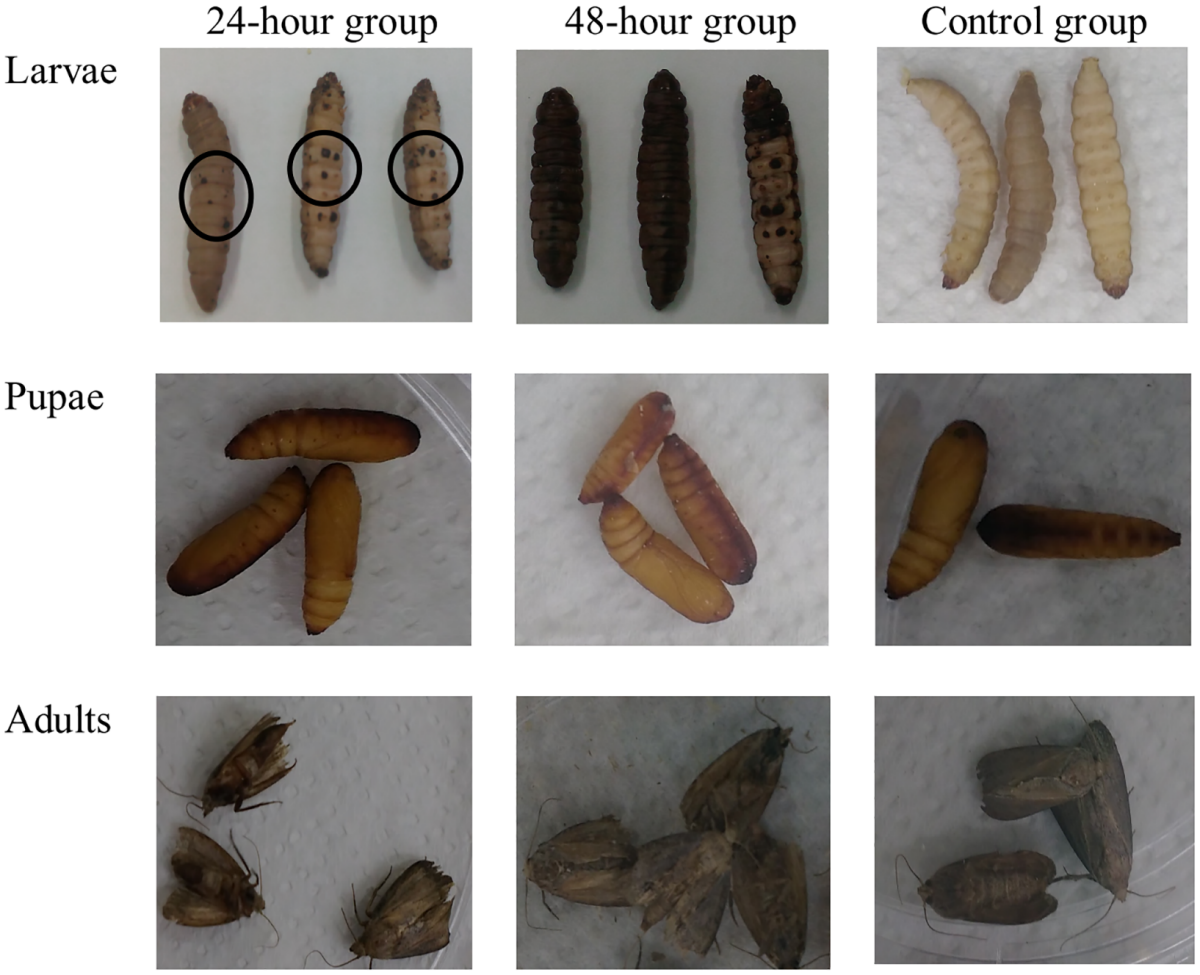
*G*. *mellonella* larvae (A), pupae (B) and adults (C) infected with the entomopathogenic fungus *C*. *coronatus* and control groups. Circles indicate changes occurring during fungal infection.

The exposure of *G*. *mellonella* larvae to sporulating colonies of *C*. *coronatus* resulted in the appearance of black spots on the cuticle of all treated insects (N = 100) at the termination of exposure. The majority of treated larvae died 24 or 48 hours following contact with the fungal pathogen (79 and 92%, respectively). In the control group formed of larvae which had no contact with the fungus, mortality was 16%. In contrast, pupae (N = 100) and adults (N = 100) were resistant, with no morphological changes observed on their cuticles after treatment (S1 Table). Fig 1 shows photographs of insects after fungal infection. However, while no adults emerged from 15% of the pupae exposed to *C*. *coronatus*, moths eclosed from all control pupae. The lifespan, behavior and mating ability of adults exposed to fungus remained unchanged.

### Enzyme activity in the cell-free filtrate of *C*. *coronatus*

The entomopathogenic fungus *C*. *coronatus* produces enzymes that are able to digest the proteins, chitin and lipids present in the insect cuticle. The proteolytic, chitinolytic and lipolytic activities of the cell-free filtrate prepared after three weeks of fungus cultivation were measured as described in the ‘Materials and methods’ section. Of the enzymes, elastase activity (55.3 ± 21.8 mM/min/mg protein) was the highest: this figure being 24 times higher than NAGase activity (2.3 ± 1.5 mM/min/mg protein; p = 0.0028, F(3,6) = 196.6), 553 times higher than chitinase activity (0.10 ± 0.04 mM/min/mg protein; p = 0.0023, F(3,6) = 258083), and 1844 times higher than lipase activity (0.03 ± 0.01 mM/min/mg protein; p = 0.0023, F(3,6) = 3411192) (S2 Table). The same cell-free filtrate was used in experiments concerning the hydrolysis of the *G*. *mellonella* cuticle, presented in this work, as well as cuticles of four medically-important fly species [20].

### Hydrolysis of the *G*. *mellonella* cuticle by fungal enzymes

**Fig 2:**
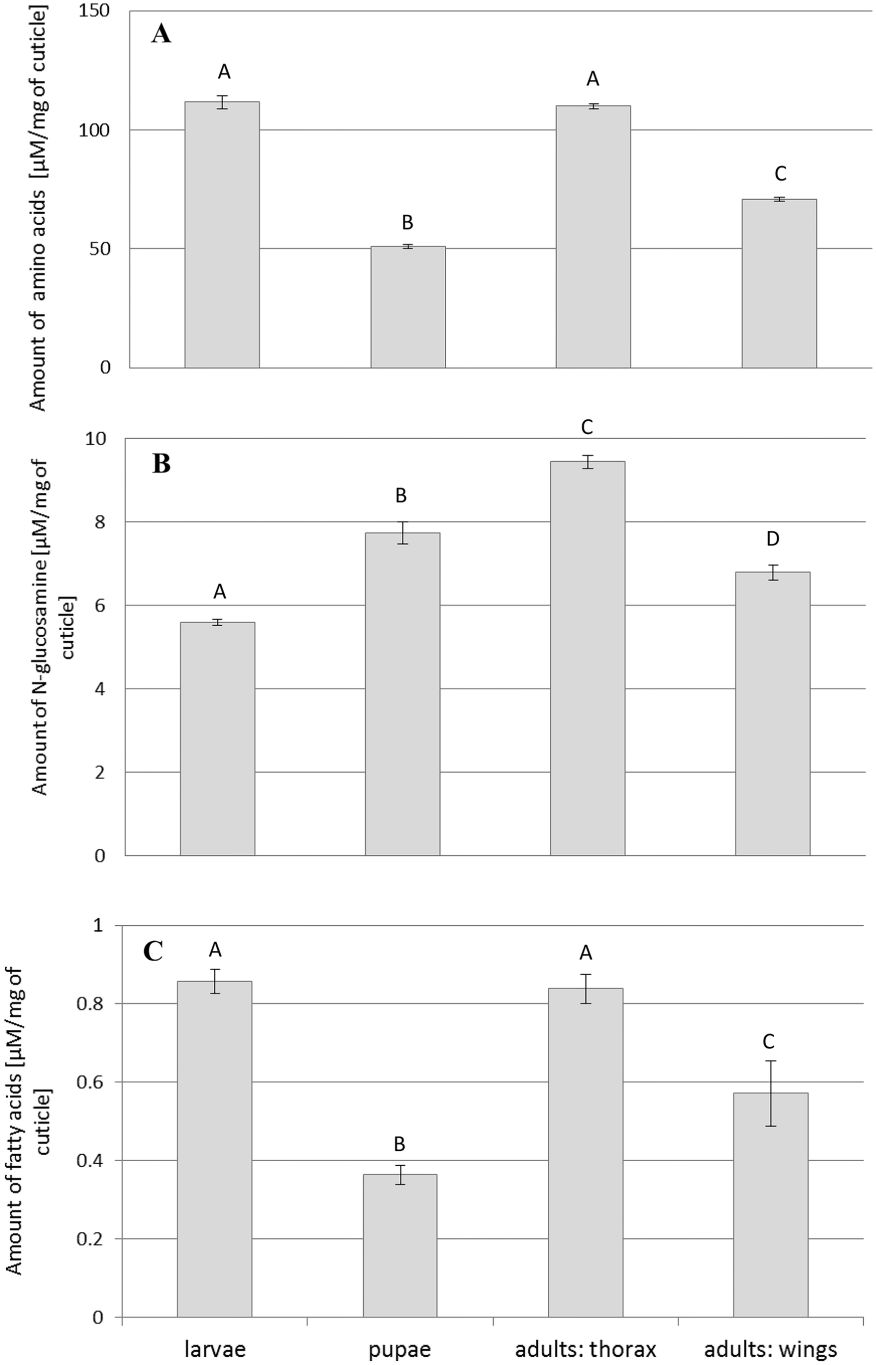
Hydrolysis of cuticular proteins (A), chitin (B) and lipids (C) by *C*. *coronatus* enzymes. Products released during 8 h of incubation are presented as mean±standard deviation μM/mg of cuticle dissected from larvae, pupae or adults (thoraces and wings) of *G*. *mellonella*.

The mixture of fungal enzymes which accumulated in the culture medium during the three-week *C*. *coronatus* cultivation was used to hydrolyze proteins present in the cuticle of *G*. *mellonella* larvae, pupae and adults. The amounts of amino acids released from the cuticle preparations during eight hours of incubation with the fungal proteases present in the cell-free filtrate are shown in Fig 2A. The highest amounts of released amino acids were measured in the digestion of the larval cuticle (111.81 μM/mg of cuticle) and adult thoraces (110.02 μM/mg of cuticle), while the lowest amount (50.85 μM/mg of cuticle) was found in the digested pupal cuticle (p<0.00001, F(3,8) = 715.13). Proteins present in the wings were digested more efficiently than those present in pupae (70.77 μM/mg of wing cuticle; p<0.0001, F(3,8) = 835).

The incubation of *G*. *mellonella* cuticle with the cell-free filtrate containing chitinases released by *C*. *coronatus* resulted in the accumulation of *N*-glucosamine, a product of chitin hydrolysis (Fig 2B). The cuticle from adult thoraces was the most susceptible to digestion by chitinases (9.44 μM/mg of cuticle) while the cuticle taken from larvae was least susceptible (5.58 μM/mg of cuticle). All observed diversities in the accumulation of *N*-glucosamine were statistically significant (p <0.001; F(3,8) = 3003)

The lipases secreted by *C*. *coronatus* hydrolyzed the lipids present in the examined cuticle samples (Fig 2C). Variations in the susceptibility to fungal lipases resembled those of cuticular proteins with regard to the stage of development (Fig 2A). Nearly equalamounts of FFA were released by the digestion of cuticle dissected from the larval (0.86 μM/mg of cuticle) and adult thoraces (0.84 μM/mg of cuticle), while less was released from the pupal cuticle (0.36 μM/mg of cuticle; p<0.001, F(3,8) = 336). The lipids present in the wings were digested more efficiently than those present in pupal cuticle (0.57 μM/mg of cuticle; p<0.001, F(2,4) = 425).

It is noteworthy that the adult wing cuticle was less efficiently digested by fungal proteases, chitinases and lipases than adult thorax cuticle (Fig 2, see also S3 Table).

### GC-MS analysis of fatty acid composition of the *G*. *mellonella* cuticle

The efficiency of cuticular lipid extraction varied according to the developmental stage of *G*. *mellonella*. Dichloromethane extraction was more efficient than ether extraction. It is noteworthy that the greatest mass of cuticular lipids was extracted from pupae (Table 1; see also S4 Table).

The cuticular lipid fractions were analyzed by GC-MS. Analysis of characteristic silyl derivative ions (M+ and M-15^+^) were used for identification the cuticular FFAs. An internal standard (19-methylarachidic acid) was applied to quantitatively determine of analyzed compounds.

Examples of the mass spectra of the trimethylsilyl (TMS) esters of hexadecanoic acid (C 16:0) and hexadecenoic acid (C 16:1) are given in S1 Fig.

S2, S3 and S4 Figs present the total ion current (TIC) of fatty acid methyl esters (after derivatization) extracted by dichloromethane from *G*. *mellonella* pupae, adults and larvae, respectively. Qualitative and quantitative GC-MS analyses of the cuticular lipids of *G*. *mellonella* showed that the identified FFAs contained from six to 20 carbon atoms in the alkyl chain.

Tables 2 and 3 (see also S5 Table) list the fatty acid content of all extracts obtained from the insects, calculated as μg/g insect body. Nine fatty acids were found in all developmental stages of *G*. *mellonella*: C8:0, C9:0, C12:0, C14:0, C15:0, C16:1, C16:0, C18:1 and C18:0. Eleven FFAs were detected in larvae, 10 in pupae and 14 in adults. C13:0, C20:1 and C20:0 were absent in larvae and pupae but present in adults. The fatty acids C16:0, C18:0 and C18:1 predominated in adults, C16:0 and C18:0 in pupae (C18:1 to a lesser extent), and C16:0 and C18:1 in larvae.

**Table 2:** Fatty acid contents in the epicuticular lipids of the *Galleria mellonella* larvae, pupae and adults (μg/g of insect body).

FFA	*Galleria mellonella* larvae			*Galleria mellonella* pupae			*Galleria mellonella* adults		
	Extract I (mean ± SD)	Extract II (mean ± SD)	Sum of FFA	Extract I (mean ± SD)	Extract II (mean ± SD)	Sum of FFA	Extract I (mean ± SD)	Extract II (mean ± SD)	Sum of FFA
**C 6:0**	6.13 ± 0.56	8.03 ± 0.87	14.16^A B^	ND	ND	ND ^A^	ND	ND	ND ^B^
**C 8:0**	4.15 ± 0.26	5.31 ± 0.45	9.46 ^C^	0.74 ± 0.01	0.71 ± 0.02	1.45 ^C^	4.28 ± 0.35	3.52 ± 0.98	7.79 ^C^
**C 9:0**	1.42 ± 0.05	4.59 ± 0.28	6.02 ^D^	1.52 ± 0.08	1.07 ± 0.11	2.59 ^D^	26.44 ± 4.18	36.13 ± 8.21	62.56^D^
**C 10:0**	1.16 ± 0.12	2.30 ± 0.41	3.47^E^	ND	ND	ND ^E^	4.70 ± 0.87	7.97 ± 1.35	12.66 ^E^
**C 12:0**	2.88 ± 0.14	5.03 ± 032	7.91	1.28 ± 0.06	1.24 ± 0.12	2.52	4.50 ± 0.45	9.85 ± 2.14	14.35
**C 13:0**	ND	ND	ND ^G^	ND	ND	ND ^F^	1.52 ± 0.14	4.78 ± 0.47	6.30 ^F G^
**C 14:0**	13.65 ± 2.17	18.09 ± 2.14	31.74 ^H^	2.16 ± 0.11	9.61 ± 1.10	11.77 ^H^	12.09 ± 2.35	26.85 ± 5.4	38.94 ^H^
**C 15:0**	4.07 ± 0.74	3.77 ± 0.87	7.84 ^I^	1.28 ± 0.04	4.26 ± 0.24	5.54 ^I K^	3.12 ± 0.41	5.57 ± 1.87	8.69 ^J K^
**C 16:1**	1.16 ± 0.08	1.74 ± 0.11	2.90 ^L^	4.19 ± 1.14	6.57 ± 0.87	10.75 ^L^	12.11 ± 3.18	21.66 ± 4.12	33.78 ^L^
**C 16:0**	136.80 ± 14.15	296.24 ± 11.15	433.04 ^M^	191.31 ± 12.36	517.73 ± 23.15	709.04 ^M^	239.78 ± 14.58	330.41 ± 12.78	570.19 ^M^
**C 17:0**	ND	ND	ND ^N^	1.40 ± 0.08	1.85 ± 0.35	3.25 ^N^	2.79 ± 0.84	4.18 ± 0.87	6.98 ^N^
**C 18:1**	136.80 ± 17.25	115.55 ± 12.15	252.35 ^O^	74.12 ± 7.15	37.86 ± 7.12	111.98 ^O^	238.38 ± 17.35	274.28 ± 13.20	512.66 ^O^
**C 18:0**	13.64 ± 3.18	25.47 ± 4.14	39.11 ^P^	186.21 ± 11.25	505.19 ± 17.54	691.40 ^P^	111.62 ± 15.12	153.67 ± 12.54	265.29 ^P^
**C 20:1**	ND	ND	ND ^R^	ND	ND	ND ^S^	30.42 ± 5.34	89.55 ± 9.35	119.97 ^RS^
**C 20:0**	ND	ND	ND ^T^	ND	ND	ND ^U^	5.85 ± 0.54	7.80 ± 1.11	13.65 ^TU^
**Sum**	321.87 ± 21.54	486.13 ± 23.45	808.00 ^W^	464.21 ± 19.45	1086.09 ± 35.36	1550.31 ^W^	697.62 ± 27.30	976.23 ± 15.35	1673.84 ^W^

FFA- free fatty acids; SD- standard deviation; Extract I- petroleum ether extract; Extract II- dichloromethane extracts; ND—not detected; statistically significant differences are marked with the same letters (ANOVA, p≤0,05), data were compared between larvae, pupae and adults

**Table 3:** Relative content (%) of fatty acids in the epicuticular lipids of the *Galleria mellonella* larvae, pupae and imago.

FFA	*Galleria mellonella* larvae			*Galleria mellonella* pupae			*Galleria mellonella* imago		
	Extract I	Extract II	Sum of FFA	Extract I	Extract II	Sum of FFA	Extract I	Extract II	Sum of FFA
**C 6:0**	1.90	1.65	1.75	ND	ND	ND	ND	ND	ND
**C 8:0**	1.29	1.09	1.17	0.16	0.07	0.09	0.61	0.36	0.47
**C 9:0**	0.44	0.95	0.74	0.33	0.10	0.17	3.79	3.70	3.74
**C 10:0**	0.36	0.47	0.43	ND	ND	ND	0.67	0.82	0.76
**C 12:0**	0.90	1.03	0.98	0.28	0.11	0.16	0.65	1.01	0.86
**C 13:0**	ND	ND	ND	ND	ND	ND	0.22	0.49	0.38
**C 14:0**	4.24	3.72	3.93	0.47	0.88	0.76	1.73	2.75	2.33
**C 15:0**	1.26	0.78	0.97	0.28	0.39	0.36	0.45	0.57	0.52
**C 16:1**	0.36	0.36	0.36	0.90	0.60	0.69	1.74	2.22	2.02
**C 16:0**	42.50	60.94	53.59	41.21	47.67	45.74	34.37	33.85	34.06
**C 17:0**	ND	ND	ND	0.30	0.17	0.21	0.40	0.43	0.42
**C 18:1**	42.50	23.77	31.23	15.97	3.49	7.22	34.17	28.10	30.63
**C 18:0**	4.24	5.24	4.84	40.11	46.51	44.60	16.00	15.74	15.85
**C 20:1**	ND	ND	ND	ND	ND	ND	4.36	9.17	7.17
**C 20:0**	ND	ND	ND	ND	ND	ND	0.84	0.80	0.82

FFA- free fatty acids; SD- standard deviation; Extract I- petroleum ether extract; Extract II- dichloromethane extract; ND—not detected

### Correlations between hydrolytic rates and cuticle composition

Table 4 presents the correlation coefficients (r) for the relationships between the concentration of each identified FFA and the effectiveness of cuticle digestion by *C*. *coronatus* proteases, chitinases and lipases. The obtained correlation coefficients varied from |0.1| to |1.0|. Scatterplots based on all analyses showed both positive and negative linear correlations with various strengths. We arbitrarily recognized the existence of a correlation between the concentration of a particular FFA in the cuticle and the effectiveness of fungal enzymes in degrading the cuticle only when a strong correlation (r ≥ 0.7 or r ≤ − 0.7) was observed in at least two developmental stages. Based on this distinction, the proteolytic degradation of the cuticle was positively correlated with C10:0, C13:0, C15:0, C17:0 and C18:0 and negatively correlated with C12:0, C16:1 and C20:1. The activities of chitinolytic and lipolytic enzymes were negatively correlated with C10:0 and C16:0.

**Table 4:** Correlation coefficients (r) the concentration of fatty acids identified in the cuticle of *G*. *mellonella* and the efficiency *of C*. *coronatus* proteases, chitinases and lipases in degrading the cuticle.

Compound	Effect on *C*. *coronatus*†	Protease				Chitinase				Lipase			
		Larvae	Pupae	Imago thorax	Imago wings	Larvae	Pupae	Imago thorax	Imago wings	Larvae	Pupae	Imago thorax	Imago wings
**C 6:0**	Negative	0.31	-	-	-	-0.94*	-	-	-	-0.99*	-	-	-
**C 8:0**	Negative	-0.98*	-0.57	-0.51	-0.66	0.45	0.79*	-0.93*	0.26	0.27	1.00*	0.23	-0.04
**C 9:0**	Negative	0.97*	-0.87*	0.42	0.58	-0.79*	0.98*	0.89*	-0.16	-0.66	0.86*	0.98*	-0.06
**C 10:0**	Negative	0.48	-	1.00*	0.98*	-0.99*	-	0.79*	-0.96*	-1.00*	-	-0.98*	0.88*
**C 12:0**	Negative	0.75*	-0.64	-0.92*	-0.83*	-0.89*	0.84*	-0.47	0.99*	-0.99*	0.99*	0.72*	-1.00*
**C 13:0**	Negative	-	-	0.84*	0.93*	-	-	0.99*	-0.67	-	-	-0.25	0.48
**C 14:0**	Negative	0.45	0.80*	-0.43	-0.59	-0.95*	-0.58	-0.90*	0.18	-0.65	0.12	0.57	0.05
**C 15:0**	Positive	0.34	0.11	0.72*	0.84*	-0.95*	0.19	1.00*	-0.51	-0.99*	0.80*	-0.99*	0.30
**C 16:1**	Negative	0.46	0.97*	-0.96*	-0.89*	-1.00*	-1.00*	-0.56	1.00*	-0.95*	-0.69	0.91*	-0.98*
**C 16:0**	Negative	0.33	-0.83*	0.81*	0.69	-0.95*	0.96*	0.27	-0.94*	-0.99*	0.90*	-0.99*	0.99*
**C 17:0**	Negative	-	0.71*	0.98*	0.92*	-	-0.47	0.63	-1.00	-	0.25	0.95*	0.96*
**C 18:1**	Negative	0.96*	-0.55	0.99*	1.00	-0.80*	0.77*	0.86*	-0.92*	-0.97*	1.00*	1.00*	0.81*
**C 18:0**	Negative	0.60	-0.57	0.96*	0.89*	-1.0*	0.79*	0.58	-1.00*	-0.98*	1.00*	-0.68	0.98*
**C 20:1**	Negative	-	-	-0.81*	-0.91*	-	-	-1.00*	0.63	-	-	-0.86*	-0.44
**C 20:0**	Negative	-	-	-0.74*	-0.61	-	-	-0.16	0.89*	*-*	-	0.94*	-0.97*

^†^- Data previously published (15);

*- high correlation

## Discussion

Entomopathogenic fungi are considered to play a vital role as biological control agents of insect populations. Although chemical pesticides are very specific in their action, most do not preserve biodiversity [21]. In contrast, a highly diverse array of fungal species is known to infect insects, and several commercial bio-insecticides based on entomopathogenic fungi have been developed. Although a number of studies have examined improvements in the production, formulation and practical application of these pesticides, much work is still needed before their final implementation. The use of entomopathogenic fungi is unavoidable as it is an fundamental part of integrated pest management programs in many ecological zones. Therefore, it is necessary to understand their underlying mechanisms of action [22].

Entomopathogenic fungi invasion begins with penetration the host cuticle. Fungal hyphae must perforate through three layers. The compounds of inner procuticle are chitin fibrils which are embedded in a proteinaceous matrix, but the outer epicuticle includes phenol-stabilized proteins. Fatty acids, lipids and sterols are the elements of waxy layer with is the outer element of the cuticle [23].

Protein and chitin elements in the insect procuticle are comparable in various insects but the epicuticular components are extremely heterogeneous. This variation may potentially result in varying responses to pathogen invasion in particular insects [8, 24].

Parasitic fungi are capable to produce a different classes of enzymes, including endoproteases, aminopetidases, carboxypetidases, N-acetylglucosoaminidases, chitinases, esterases and lipases, which determine fungal virulence [3,4].

Our findings show that the entomopathogenic fungus *C*. *coronatus* produces chitinolytic, lipolytic and proteolytic enzymes that hydrolyze *in vitro* the cuticle of *G*. *mellonella*. Other authors have also shown that the fungus produces several proteolytic enzymes: 6.8-kDa alkaline serine proteinase [25], 23-kDa proteinase I, and 19-kDa proteinase II, which are the products of autoproteolysis of proteinase I [26], 27- to 28.5-kDa subtilisin-like proteinase, and a novel 30- to 32-kDa subtilisine-like serine protease [27].

EST (expressed sequence tags) analysis confirms our finding that *C*. *coronatus* produces a chitinolytic complex to degrade cuticular components [27,28]. Lipolytic activity in *C*. *coronatus* cultures has also been reported [20, 29].

St. Leger et al. [30] demonstrated that *M*. *anisopliae* produces proteases which hydrolyze the cuticles of *Caliphora vomitoria* and *Manduca sexta*. *Beauveria bassiana* produces protease, amylase, caseinase, chitinase and lipase against *Helicoverpa armigera* [31].

The digestion of protein substrates by peptidases is required for infection. Two types of enzymes can be used for this process: endopeptidases cleave peptide bonds inside the peptide molecule whereas exopeptidases degrade bonds at the end of the protein chain. The best known determinant of fungal entomopathogenicity is the subtilisin-like serine protease Pr1 of *Metarhizium anisopliae*: a protein with four isoforms whose role in host invasion has been clearly demonstrated. Fungal hyphae produce this enzyme with the formation of the appressorium and at the time of penetration. A trypsin-like enzyme (Pr2) has been observed during the early stages of cuticle colonisation, which suggests that it may play some role in degrading the host proteins [30].

Chitinases hydrolyse the linear polymer chitin, a polysaccharide of β (1,4) linked *N-*acetylglucosamine (GlcNAc; 2-acetamino-2-deoxy-β-Dglucose) units [32]. *N*-acetyloglucosoaminidase (NAGase) is one of the enzymes which participate in that process. Chitinolytic enzymes have been identified in many species of entomopathogenic fungi, with the best examples being described in *M*. *anisopliae*: a 33 kDachitinase, 110 kDaβNacetyloglucoamidase [33], a 60 kDa protein with exo- and endochitinase activity [34], a 30 kDachitinase and two chitinases 43,5 kDa and45 kDa [33]. Chitinases are very widespread in the natural world, and other studies have been identified in insects, crustaceans, yeasts and fungi, as well as in bacteria and vertebrates. Fungi produce chitinases for building cell membranes, and are employed in germination, sprouting hyphae and autolysis [30].

The final set of enzymes are lipases, defined as triacylglycerol acyl hydrolases (EC 3.1.1.3). The best-known lipases are those produced by *Rhizopus sp*., *Penicillium sp*., *Aspergillus sp*., *Mucor sp*., *Ashbya sp*., *Geotrichum sp*., *Beauveria sp*., *Humicola sp*., *Rhizomucor sp*., *Fusarium sp*., *Acremonium sp*., *Alternaria sp*., *Eurotrium sp*., *Ophiostoma sp*., [35].

The present work clearly indicates the differences in the hydrolysis of cuticle material from *G*. *mellonella* larvae, pupae, adult thorax and wings by enzymes produced by *C*. *coronatus*. Cuticle portions dissected from larvae and adult thorax were the most susceptible to digestion by proteases and lipases, and the maximum concentration of free *N*-glucosamine was recorded in the case of hydrolyzed moth thorax. Differences in the products of fungal enzyme digestion of cuticles taken from various developmental stages may reflect stage-specific differences in cuticle composition, as confirmed by GC/MS examinations of the developmental changes occurring in the FFA profiles: The FFA profile of *G*. *mellonella* larvae was composed of 11 FFAs, while the cuticular profiles of pupae and adults contained 10 and 14 FFAs, respectively. Nine fatty acids were found in the cuticle of all developmental stages of *G*. *mellonella*: C8:0, C9:0, C12:0, C14:0, C15:0, C16:1, C16:0, C18:1 and C18:0. Three FFAs C13:0, C20:0 and C20:1 were present only in adults, being absent in larvae and pupae. Four FFAs C13:0, C17:0, C20:0 and C20:1 were found only in pupae and adults, while absent in larvae.

The qualitative and quantitative stage-related differences in the FFA profiles of the *G*. *mellonella* cuticle presented in Tables 2 and 3 indicate the profound developmental changes occurring in lipid management. The mechanisms underlying these changes remain obscure. The presence and amount of each cuticular FFA is the resultant of multistep hitherto unexplored processes of synthesis, degradation as well as distribution in the insect body and transportation to the target sites. Other reviews explore the role of the cuticle in fostering multilevel relationships between insects, where it acts as a source of chemical signaling [36]. Larvae, pupae and adults have widely differing roles in the wax moth population, and so the information they use to communicate with other individuals, based on cuticle lipids, is also different.

According to our findings, the unsaturated acids C16:1 and C18:1 were found in the cuticles from *G*. *mellonella* all stages, while C20:1 were detected only in that from the imago. These FFAs were also identified in the cuticular lipids of adult *S*. *gregaria* [37] and in the pupae and larvae of *Caliphora vicina* [38].

Free fatty acids with molecular weights between 21:0 and 26:0 were not detected in our study. They are present in the cuticle of *C*. *vicina* but only in trace amounts [38]. The present work clearly highlights the qualitative and quantitative differences in cuticular lipid components between the *G*. *mellonella* larvae, pupae and imagines. Statistically significant differences (p≤0.05) were found between the pupae and larvae for each detected fatty acid.

A previous analysis of the cuticular lipids from *G*. *mellonella* larvae confirmed the presence of C16:0, C18:0, C18:1 and C18:2, as well as traces of C14:0, C16:1, C20:0 and C20:1 [38]. While the present analysis, performed using a different methodology and GC/MS apparatus, failed to confirm the presence of C18:2 in the larval cuticle, it did detect the presence of other FFAs.

Stage-specific profiles of cuticular FFAs have been reported in two other insect species. In *Lasioderma serricorne*, the larval cuticle contains C20:0, C22:0 and C24:0 but the adult cuticle contains only C20:0 and C22:0 [39]. In another study, based on *C*. *vicina*, 16 saturated, five monounsaturated, one diunsaturated and two polyunsaturated FFAs were found in larval cuticles, while 18 saturated, nine monounsaturated, two diunsaturated and three polyunsaturated FFAs were found in pupal cuticles. The major cuticular FFAs in *C*. *vicina* larvae and pupae was C18:1. The larvae were found to contain greater amounts of total cuticular FFAs (1.7 mg/g of the insect body) than the pupae (1.4 mg/g of the insect body) [38].

The significant differences observed between the moth wing and thoracic cuticles regarding the efficiency of degradation of proteins, chitin and lipids suggest the presence of differences in the chemical compositions and architectures of these adult structures, and in the spatial distribution of their constituent components. However, further experiments are needed to confirm the spatial distribution of cuticular FFAs.

Fatty acids present in the insects cuticle have many important biological functions. Along with other cuticular lipids they protect insects from water loss and hence preventing lethal desiccation [40]. They are also the first barrier against chemical or biological contact insecticides [41]. Esterified fatty acids from honeybee larvae were reported to act as pheromones for honeybee workers and also as kairomones for parasitic mites [42]. The C16 and C18 acids were reported to participate in the chemical camouflage of a moth (*Acherontiaatropos L*) in honeybee colonies [43]. Our findings highlight the great diversity in susceptibility to fungal infection demonstrated by larvae, pupae and adults. This is probably related to the very large qualitative and quantitative differences present in the cuticle fatty acid in each examined developmental stage. Boguś at al. [15] examined the effects of fatty acids which were found in insect cuticle on the *in vitro* growth and pathogenicity of the entomopathogenic fungus *C*. *coronatus* (the same strain that was used in our tests). At high concentrations, spore germination was inhibited by C7:0, C8:0, C9:0, C10:0, C12:0, C18:2 and C18:3, while hyphal growth was block out by C5:0, C6:0, C6:2, C14:0, C16:0, C16:1, C18:0, C18:1, C20:0 and C20:1. The presence of 0.1% C15:0 stimulated the growth of *C*. *coronatus* on LB and MM medium. Sporulation was inhibited by all concentrations (0.1–0.0001%) of C16:0 and C18-20 fatty acids. Sporulation was stimulated by low concentrations of C5:0, C6:0, C6:2 and C7:0. Fatty acids C5-12, as well as C18:3, C20:0 and C20:1 retarded the fungal penetration of *G*. *mellonella* while C16:1 increased infection. It suggest that C16:1 may potentiate the production of enzymes involved in the invasion. The toxicity of metabolites released into the incubation medium decreased by varying degrees in the presence of C6:0, C6:2, C7:0, C9:0, C12:0, C16:1, C18:2, C18:3, C20:0 and C20:1. No such effect was observed for other tested fatty acids.

The fatty acids present in insect cuticles have an impact on the virulence of the entomopathogenic fungi, including the activity of enzymes digesting the host cuticle. Our results show that the fatty acids present in the cuticle may modulate the activity of fungal enzymes. The proteolytic degradation of the cuticle was positively correlated with C10:0, C13:0, C15:0, C17:0 and C18:0 and negatively correlated with C12:0, C16:1 and C20:1. The activities of chitinolytic and lipolytic enzymes were negatively correlated with C10:0 and C16:0.

These cuticle components have been implicated in the inhibition of fungal propagule germination [44]. Fatty acids (short-chain saturated) from *Heliotiszea* can decrease the growth of *Beaveria bassiana* [45]. According to Uziel and Kenneth [46], C14:0 and C18:1 stimulate germ-tube formation in two *Erynia* species of the Entomophthorales (*E*. *delphacis* and *E*. *neoaphidis*), while C18:2 was fungistatic to most tested conidia of both species. The cuticular fatty acids of the housefly *Fannia canicularis* regulate the germination of *Euchalcia variabilis* conidia: C18:1 induce germination, high levels of C16:1 stimulate the fungal mycelia but have a negative impact on conidia, while low levels of C18:2 and C18:3 have damaging effects on spore germination and mycelial proliferation. Barnes and Moore [47] note that C8:0 and C10:0 inhibit the germination of *M*. *flavovoride*. High concentrations of C18:0 can have a positive effect. In addition, C18:0 and C12:0 could stimulate the germination of *M*. *flavovoride* conidia.

The virulence of entomopathogenic fungi consists of two stages. First the growing hyphae perforate the insect cuticle, then come up to the enzymatic degradation of major cuticle chemical components, i.e. proteins, chitin, and lipids. As insect cuticular fatty acids influence the *in vitro* growth and pathogenicity of the entomopathogenic fungi, the qualitative and quantitative differences in the FFA profiles observed in the cuticle of *G*. *mellonella* at various developmental stages may be responsible for the heterogeneous efficiency of fungal enzymes in degrading larval, pupal and adult cuticles, and hence the varied sensitivity of insects to fungal infection. A deeper knowledge of the role played by cuticular lipids in the interaction between the invading fungus with the insect host allow to understand the infection of entomopathogenic fungi.

## Supporting information

S1 Table The sensitivity of insects to fungal infection. (XLSX)

S2 Table Enzyme activity in the cell-free filtrate of *C*. *coronatus*. (XLS)

S3 Table Hydrolysis of the G. mellonella cuticle by fungal enzymes- raw data. (XLSX)

S4 Table Quantitative summary of the experiment- raw data. (XLSX)

S5 Table Fatty acid contents in the epicuticular lipids of the *Galleria mellonella* larvae, pupae and adults- raw data. (XLSX)

S1 Fig Mass spectrum of the trimethylsilyl (TMS) ester of hexadecenoic acid (A) and hexadecanoic acid (B)- the compounds found in the samples. (TIF)

S2 Fig The total ion current (TIC) of fatty acids (TMS esters) of the dichloromethane extract from *G*. *mellonella* pupae. (IS- internal standard- 19-methylarachidic acid); fatty acids and molecular ions: 1- octanoic acid (C 8:0, m/z = 201); 2- nonanoic acid (C 9:0, m/z = 215); 3- dodecanoic acid (C 12:0, m/z = 257); 4- tetradecanoic acid (C 14:0, m/z = 285); 5- pentadecanoic acid (C 15:0, m/z = 299); 6- hexadecenoic acid (C 16:1, m/z = 311); 7- hexadecanoic acid (C 16:0, m/z = 313); 8- heptadecanoic acid (C 17:0, m/z = 327); 9- octadecenoic acid (C 18:1, m/z = 339); 10- heptadecanoic acid (C 18:0, m/z = 341); 11- eicosanoic acid (C 20:0, m/z = 369).
(TIF)

S3 Fig The total ion current (TIC) of fatty acids (TMS esters) of the dichloromethane extract from *G*. *mellonella* adults. (IS- internal standard- 19-methylarachidic acid); fatty acids and molecular ions: 1- octanoic acid (C 8:0, m/z = 201); 2- nonanoic acid (C 9:0, m/z = 215); 3- decanoic acid (C 10:0, m/z = 229); 4- dodecanoic acid (C 12:0, m/z = 257); 5- tridecanoic acid (C 13:0, m/z = 271); 6- tetradecanoic acid (C 14:0, m/z = 285); 7- pentadecanoic acid (C 15:0, m/z = 299); 8- hexadecenoic acid (C 16:1, m/z = 311); 9- hexadecanoic acid (C 16:0, m/z = 313); 10- heptadecanoic acid (C 17:0, m/z = 327); 11- octadecenoic acid (C 18:1, m/z = 339); 12- heptadecanoic acid (C 18:0, m/z = 341); 13- eicosenoic acid (C 20:1, m/z = 367); 14- eicosanoic acid (C 20:0, m/z = 369).
(TIF)

S4 Fig The total ion current (TIC) of fatty acids (TMS esters) of the dichloromethane extract from *G*. *mellonella* larvae. (IS- internal standard- 19-methylarachidic acid); fatty acids and molecular ions: 1- hexanoic acid (C 6:0, m/z = 173); 2- octanoic acid (C 8:0, m/z = 201); 3- nonanoic acid (C 9:0, m/z = 215); 4- decanoic acid (C 10:0, m/z = 229); 5- dodecanoic acid (C 12:0, m/z = 257); 6- tetradecanoic acid (C 14:0, m/z = 285); 7- pentadecanoic acid (C 15:0, m/z = 299); 8- hexadecenoic acid (C 16:1, m/z = 311); 9- hexadecanoic acid (C 16:0, m/z = 313); 10- octadecenoic acid (C 18:1, m/z = 339); 11- heptadecanoic acid (C 18:0, m/z = 341).
(TIF)
