# Supplementary figures and images for: Cuticular fatty acids of Galleria mellonella (Lepidoptera) inhibit fungal enzymatic activities of pathogenic Conidiobolus coronatus
Source: PLoS One. 2018 Mar 8;13(3):e0192715. doi: 10.1371/journal.pone.0192715 (PMC5843172; doi:10.1371/journal.pone.0192715)

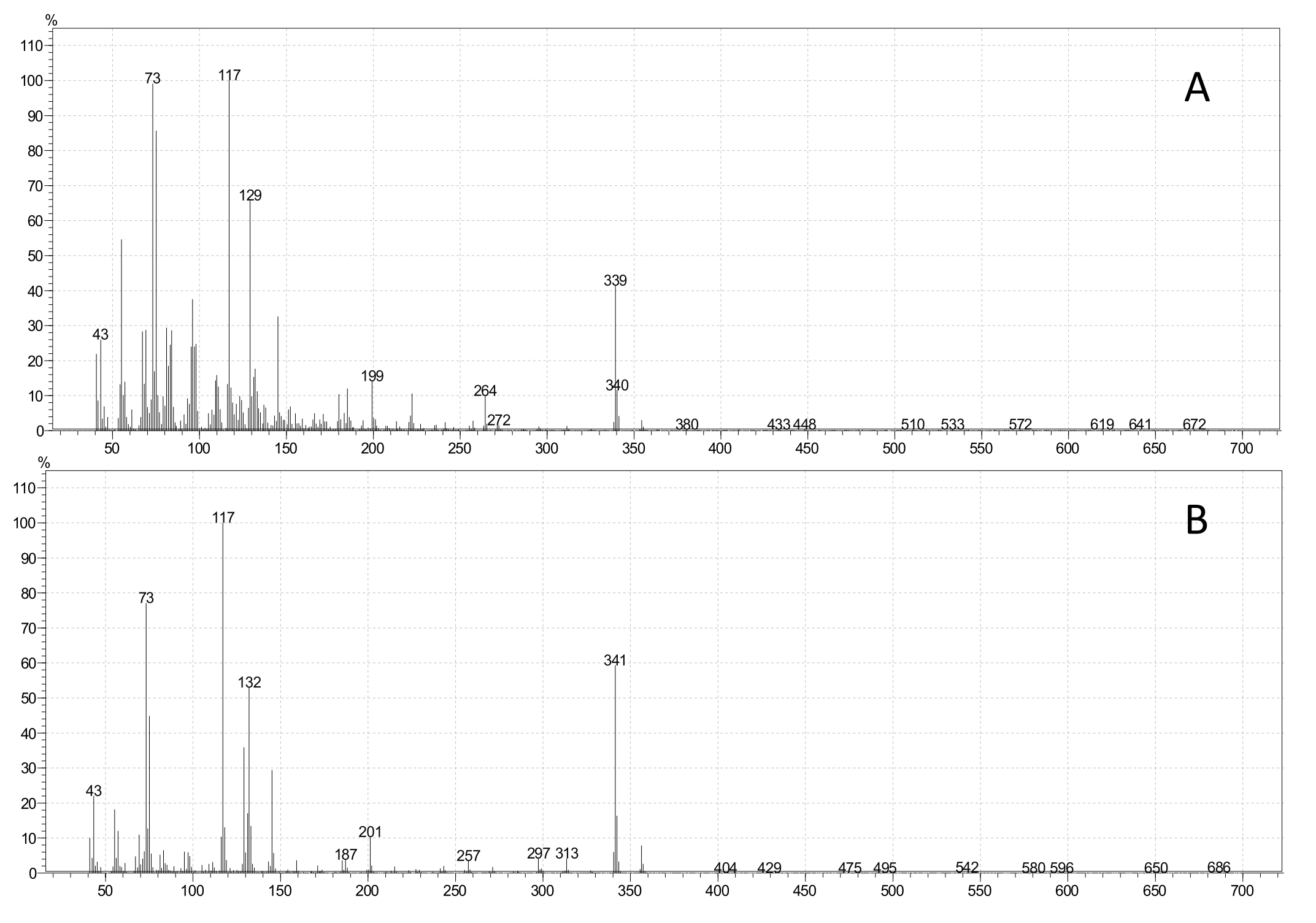

Supplement: S1 Fig — (TIF) [file pone.0192715.s006.tif]

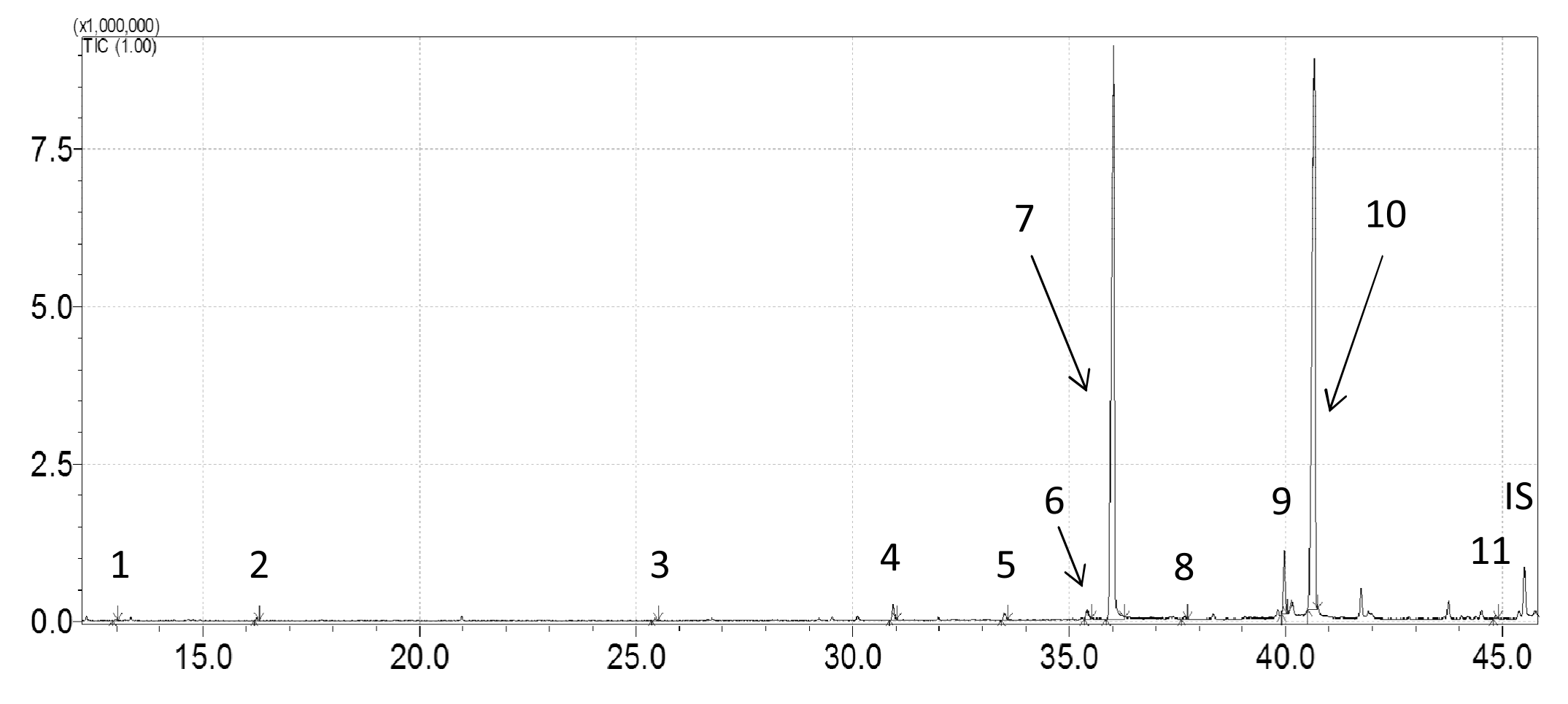

Supplement: S2 Fig — (IS- internal standard- 19-methylarachidic acid); fatty acids and molecular ions: 1- octanoic acid (C 8:0, m/z = 201); 2- nonanoic acid (C 9:0, m/z = 215); 3- dodecanoic acid (C 12:0, m/z = 257); 4- tetradecanoic acid (C 14:0, m/z = 285); 5- pentadecanoic acid (C 15:0, m/z = 299); 6- hexadecenoic acid (C 16:1, m/z = 311); 7- hexadecanoic acid (C 16:0, m/z = 313); 8- heptadecanoic acid (C 17:0, m/z = 327); 9- octadecenoic acid (C 18:1, m/z = 339); 10- heptadecanoic acid (C 18:0, m/z = 341); 11- eicosanoic acid (C 20:0, m/z = 369). (TIF) [file pone.0192715.s007.tif]

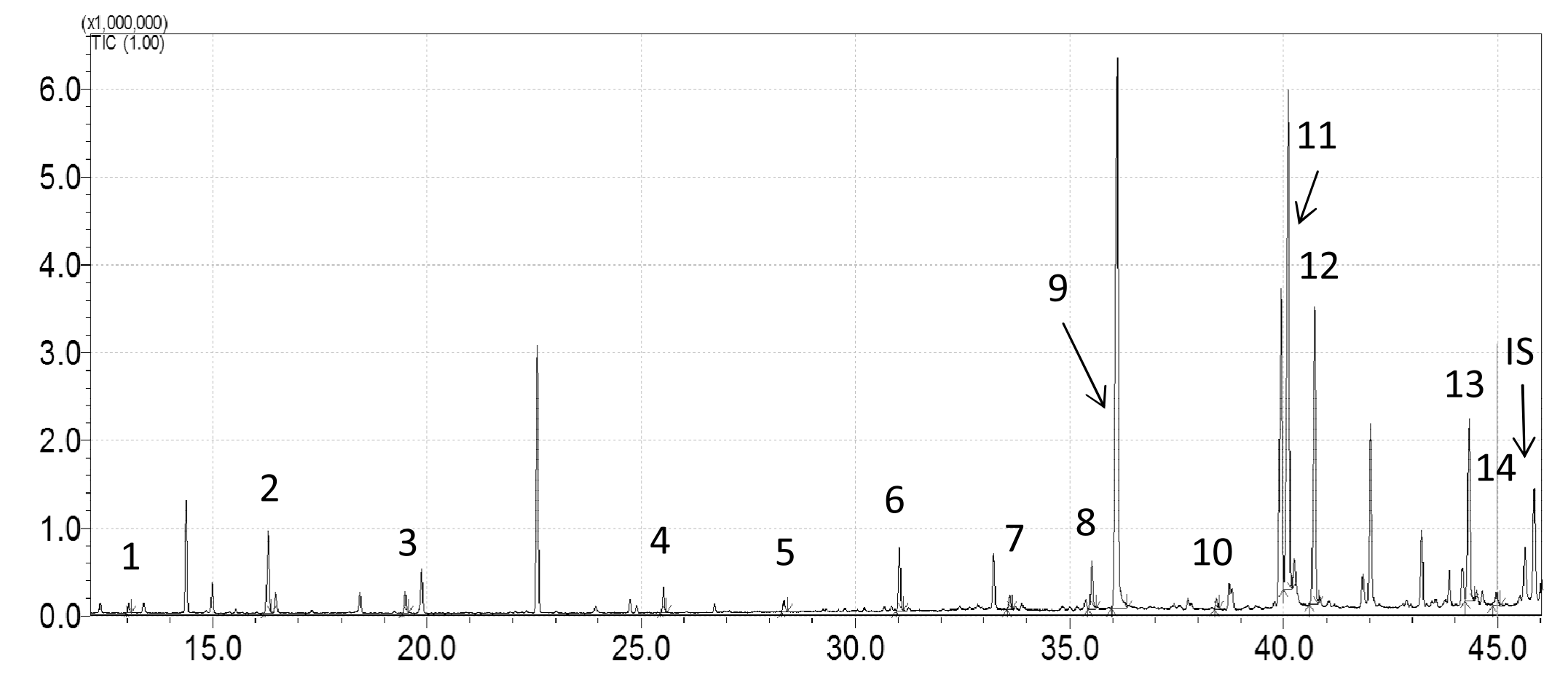

Supplement: S3 Fig — (IS- internal standard- 19-methylarachidic acid); fatty acids and molecular ions: 1- octanoic acid (C 8:0, m/z = 201); 2- nonanoic acid (C 9:0, m/z = 215); 3- decanoic acid (C 10:0, m/z = 229); 4- dodecanoic acid (C 12:0, m/z = 257); 5- tridecanoic acid (C 13:0, m/z = 271); 6- tetradecanoic acid (C 14:0, m/z = 285); 7- pentadecanoic acid (C 15:0, m/z = 299); 8- hexadecenoic acid (C 16:1, m/z = 311); 9- hexadecanoic acid (C 16:0, m/z = 313); 10- heptadecanoic acid (C 17:0, m/z = 327); 11- octadecenoic acid (C 18:1, m/z = 339); 12- heptadecanoic acid (C 18:0, m/z = 341); 13- eicosenoic acid (C 20:1, m/z = 367); 14- eicosanoic acid (C 20:0, m/z = 369). (TIF) [file pone.0192715.s008.tif]

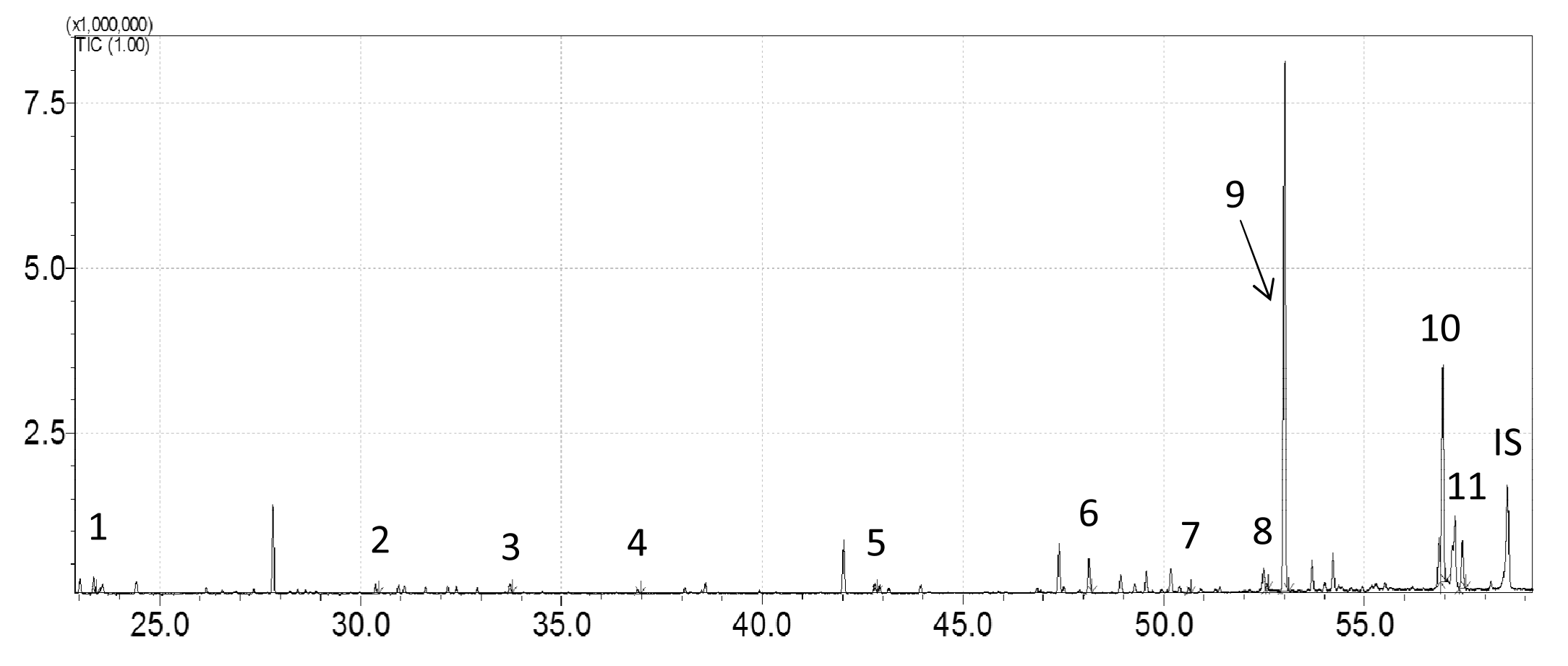

Supplement: S4 Fig — (IS- internal standard- 19-methylarachidic acid); fatty acids and molecular ions: 1- hexanoic acid (C 6:0, m/z = 173); 2- octanoic acid (C 8:0, m/z = 201); 3- nonanoic acid (C 9:0, m/z = 215); 4- decanoic acid (C 10:0, m/z = 229); 5- dodecanoic acid (C 12:0, m/z = 257); 6- tetradecanoic acid (C 14:0, m/z = 285); 7- pentadecanoic acid (C 15:0, m/z = 299); 8- hexadecenoic acid (C 16:1, m/z = 311); 9- hexadecanoic acid (C 16:0, m/z = 313); 10- octadecenoic acid (C 18:1, m/z = 339); 11- heptadecanoic acid (C 18:0, m/z = 341). (TIF) [file pone.0192715.s009.tif]
